# Beta-blockers, depression, and anxiety after myocardial infarction: the BETAMI-DANBLOCK trial

**DOI:** 10.1093/eurheartj/ehag200

**Published:** 2026-03-25

**Authors:** Johanne E Nyen, Anna Meta D Kristensen, Toril Dammen, Ann-Dorthe Zwisler, Michael Hecht Olsen, Michael Maeng, Sigrun Halvorsen, Kjell Vikenes, Arnhild Bakken, Therese Holmager, Morten W Fagerland, Dan Atar, Eva Prescott, John Munkhaugen

**Affiliations:** Department of Medicine, Drammen Hospital, Vestre Viken Hospital Trust, Jacob Borchs Gate 25, Drammen 3012, Norway; Department of Behavioral Medicine, Institute of Basic Medical Sciences, Faculty of Medicine, University of Oslo, Oslo, Norway; Department of Cardiology, Copenhagen University Hospital–Bispebjerg and Frederiksberg, Copenhagen, Denmark; Division of Mental Health and Addiction, Department of Research and Innovation, Oslo University Hospital, Oslo, Norway; Institute of Clinical Medicine, Faculty of Medicine, University of Oslo, Oslo, Norway; Department of Oncology, Clinic for Palliative Medicine, Rehabilitation and Patient-Centered Care, Copenhagen, Denmark; Department of Clinical Medicine, Faculty of Health and Medical Sciences, University of Copenhagen, Copenhagen, Denmark; Department of Clinical Medicine, University of Copenhagen, Copenhagen, Denmark; Department of Internal Medicine 1, Holbæk Hospital, Holbæk, Denmark; Department of Cardiology, Aarhus University Hospital, Aarhus University, Aarhus, Denmark; Department of Clinical Medicine, Aarhus University, Aarhus, Denmark; Institute of Clinical Medicine, Faculty of Medicine, University of Oslo, Oslo, Norway; Department of Cardiology, Oslo University Hospital Ullevaal, Oslo, Norway; Department of Heart Disease, Haukeland University Hospital and University of Bergen, Bergen, Norway; Department of Cardiology, Oslo University Hospital Ullevaal, Oslo, Norway; Department of Cardiology, Copenhagen University Hospital–Bispebjerg and Frederiksberg, Copenhagen, Denmark; Oslo Centre for Biostatistics and Epidemiology, Research Support Services, Oslo University Hospital, Oslo, Norway; Institute of Clinical Medicine, Faculty of Medicine, University of Oslo, Oslo, Norway; Department of Cardiology, Oslo University Hospital Ullevaal, Oslo, Norway; Department of Cardiology, Copenhagen University Hospital–Bispebjerg and Frederiksberg, Copenhagen, Denmark; Department of Medicine, Drammen Hospital, Vestre Viken Hospital Trust, Jacob Borchs Gate 25, Drammen 3012, Norway; Department of Behavioral Medicine, Institute of Basic Medical Sciences, Faculty of Medicine, University of Oslo, Oslo, Norway

**Keywords:** Beta-blocker, Myocardial infarction, Side effects, Randomized trial, Depression, Anxiety

## Abstract

**Structured Graphical Abstract:**
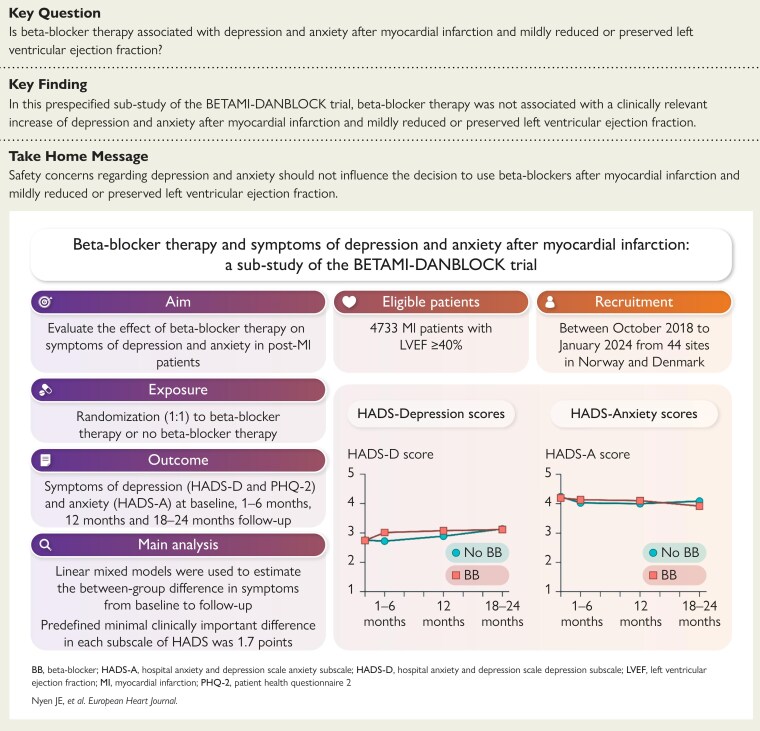

**See the editorial comment for this article ‘When are beta-blockers safe to use? Symptoms of depression and anxiety in aggregated data of pragmatic open-label trials’, by P. Leissner, https://doi.org/10.1093/eurheartj/ehag296.**

## Introduction

Symptoms of depression and anxiety are common among patients with myocardial infarction (MI) and associated with reduced health-related quality of life and increased risk of recurrent major adverse cardiovascular events.^1,2^ The underlying causes of post-MI depression and anxiety are still a matter of elucidation.^3^ They may be pre-existing conditions caused by environmental, biological or genetic factors, as well as a response to the MI event itself and/or side effects of the treatments initiated.^3^

Beta-blocker therapy has been a cornerstone in secondary prevention following MI, based on trials conducted several decades ago documenting their beneficial effects on morbidity and mortality. Current guidelines for the management of patients with acute coronary syndrome support the use of beta-blockers for all post-MI patients.^4,5^ However, major advancements in the acute and long-term MI treatment with coronary revascularization and antithrombotic, lipid-lowering, and blood pressure-lowering drugs have prompted a re-evaluation of the role of beta-blockers in modern clinical practice.^6^ Recently, the REDUCE-AMI^7^ and REBOOT^8^ trials on MI patients without heart failure found no effect of beta-blocker therapy on their primary composite endpoints. In contrast, the BETAMI-DANBLOCK^9^ and ABYSS^10^ trials, also in patients without heart failure, found a beneficial effect of beta-blocker therapy on a primary composite endpoint of all-cause mortality and major adverse cardiovascular events.

For a full assessment of the benefits and risks of beta-blockers, it is also necessary to consider the possible side effects. Beta-blocker therapy has been associated with neuropsychological side effects, including depression, anxiety, fatigue, and insomnia.^11–15^

The aim of this study was to investigate the effect of beta-blocker therapy on symptoms of depression and anxiety in post-MI-patients in the BETAMI-DANBLOCK trial.^16^

## Methods

### Trial design and population

This was a prespecified sub-study of the joint BETAMI-DANBLOCK trial. BETAMI and DANBLOCK were multicentre, open-label randomized controlled trials conducted at 19 sites in Norway and 25 sites in Denmark designed to evaluate the effects of beta-blocker therapy after MI in patients with mildly reduced or preserved left ventricular ejection fraction (LVEF).^16^ The trials had almost identical designs and criteria for inclusion and exclusion. The protocols were harmonized from the time of trial design in order to allow us to conduct joint analyses based on pooled data. Further details on the design of the trials are described in previous publications.^9,16^ The trials were conducted in accordance with ICH-GCP Guidelines, approved by the relevant ethics committees and registered at ClinicalTrials.gov (identifiers NCT03778554, NCT03646357).

Patients who provided written informed consent within 14 days after an MI with mildly reduced or preserved LVEF (≥40%) were eligible for participation. Exclusion criteria were medical conditions requiring or contraindicating beta-blocker therapy, e.g. clinical signs of heart failure. The trial design and additional inclusion and exclusion criteria have been described in detail previously.^9,16^

Clinical data, registry data, and patient-reported outcome measures (PROMs) including the Hospital Anxiety and Depression Scale (HADS) and the Patient Health Questionnaire-2 (PHQ-2) (BETAMI cohort only) were collected before randomization and throughout the follow-up period. All patients included were invited to complete PROMs and a self-reported screening question concerning adherence to their allocated treatment (described in the *Appendix*), either electronically or on paper, at 1-, 6-, 12-, and 18-months follow-up in BETAMI and at 3-, 12-, and 24-months follow-up in DANBLOCK. Patients who did not complete any PROMs during the trial period were excluded from this sub-study.

### Randomization and treatment

Participants were randomized 1:1 stratified on trial sites (BETAMI) and LVEF (DANBLOCK), to receive usual care including a beta-blocker, or usual care without a beta-blocker. Dose and type of beta-blocker were at the discretion of the treating physician, but physicians were encouraged to give the highest tolerated dose. Patients randomized to no beta-blocker therapy while on beta-blockers at the index date had their beta-blockers discontinued by the treating physician as soon as clinically possible.

### Outcome

The main outcome of this sub-study was the between-group difference in changes in depression (HADS-D) score from baseline to 12-month follow-up. Secondary outcomes were between-group difference in changes in anxiety scores (HADS-A), depression scores assessed by PHQ-2, and cases of depression and anxiety.

HADS is a validated questionnaire for assessing symptoms of depression and anxiety.^17^ It comprises 14 items, with seven assessing depressive symptoms (HADS-D) and seven assessing anxiety symptoms (HADS-A), each rated on a 4-point Likert scale.^18^ Summarizing the responses within each subscale yields separate scores for depression and anxiety, each ranging from 0 to 21.^18^ Based on prior studies in patients with cardiovascular disease, a minimal clinically important difference of 1.7 points was used to interpret between-group differences in HADS-D and HADS-A.^19^

HADS-D or HADS-A scores of eight or more are considered indicative of clinically significant symptoms of depression or anxiety.^17^

Symptoms of depression were also measured with PHQ-2 in the BETAMI-cohort. The questionnaire consists of two questions about the level of depressed mood and anhedonia over the past two weeks.^20^ The maximum score is six, with a score of three or more considered an indicator of potential clinical depression.^21,22^

### Statistical analysis

The number of patients included in BETAMI-DANBLOCK was determined based on power calculation for the primary composite endpoint of all-cause death or major adverse cardiovascular events.^9,16^

Time of completed PROMs is stated as number of months since baseline. Due to different follow-up intervals in BETAMI and DANBLOCK, time points are in the joint analyses classified in the following categories: baseline, 1–6 months from baseline, 12 months from baseline, and 18–24 months from baseline. For patients who completed several PROMs within a time window (i.e. both at 1 and 6 months in the BETAMI-cohort), the mean PROM-score in the time window was applied. The original time points are applied for analyses conducted by country.

Analyses were prespecified and performed on the intention-to-treat population. The sub-group of doses ≥100 mg/day was added *post hoc*. Linear mixed models were applied to evaluate the between-group differences in changes in scores from baseline to each time point: 1–6 months, 12 months, and 18–24 months. The dependent variables were the HADS-D score, HADS-A score, and PHQ-2 score. The linear mixed models included fixed effects for treatment (beta-blocker vs no beta-blocker), time point (baseline, 1–6 months, 12 months, and 18–24 months), treatment × time interaction, and the factors used to stratify the randomization (LVEF over 50% and trial sites). The interaction term between treatment and time was included to estimate between-group differences [with 95% confidence intervals (CI)] in change over time. Additionally, patient-level random effects were included to account for individual variability within the trial population. The effect of beta-blockers on clinically significant symptoms of depression and anxiety was estimated as odds ratios (OR) in multivariable logistic regressions. The dependent variables were dichotomous variables for clinically significant symptoms of depression or anxiety (HADS-D or HADS-A scores ≥8), and the effect of beta-blocker therapy over time was identified by an interaction term between beta-blocker group and follow-up point. All analyses were adjusted for the stratification variables LVEF and trial site.

A sensitivity analysis was conducted in which patients were categorized into either the beta-blocker group or no beta-blocker group based on self-reported adherence at each follow-up. Subgroup analyses were performed to examine the outcomes stratified by baseline characteristics, including HADS-D and HADS-A scores (<8 vs ≥8), sex (female vs male), age (<70 vs ≥70 years), country (Norway vs Denmark), LVEF (≥50% vs <50%), beta-blocker doses (≥ vs <median dose), and prior beta-blocker therapy. An exploratory analysis estimated the between-group differences in changes in each of the 14 items of HADS and the two items of PHQ-2, to reveal if specific items were affected by beta-blocker therapy.

Analyses were performed using R version 4.4.2 (R Foundation, Vienna, Austria).

## Results

### Patient characteristics

**Figure 1:**
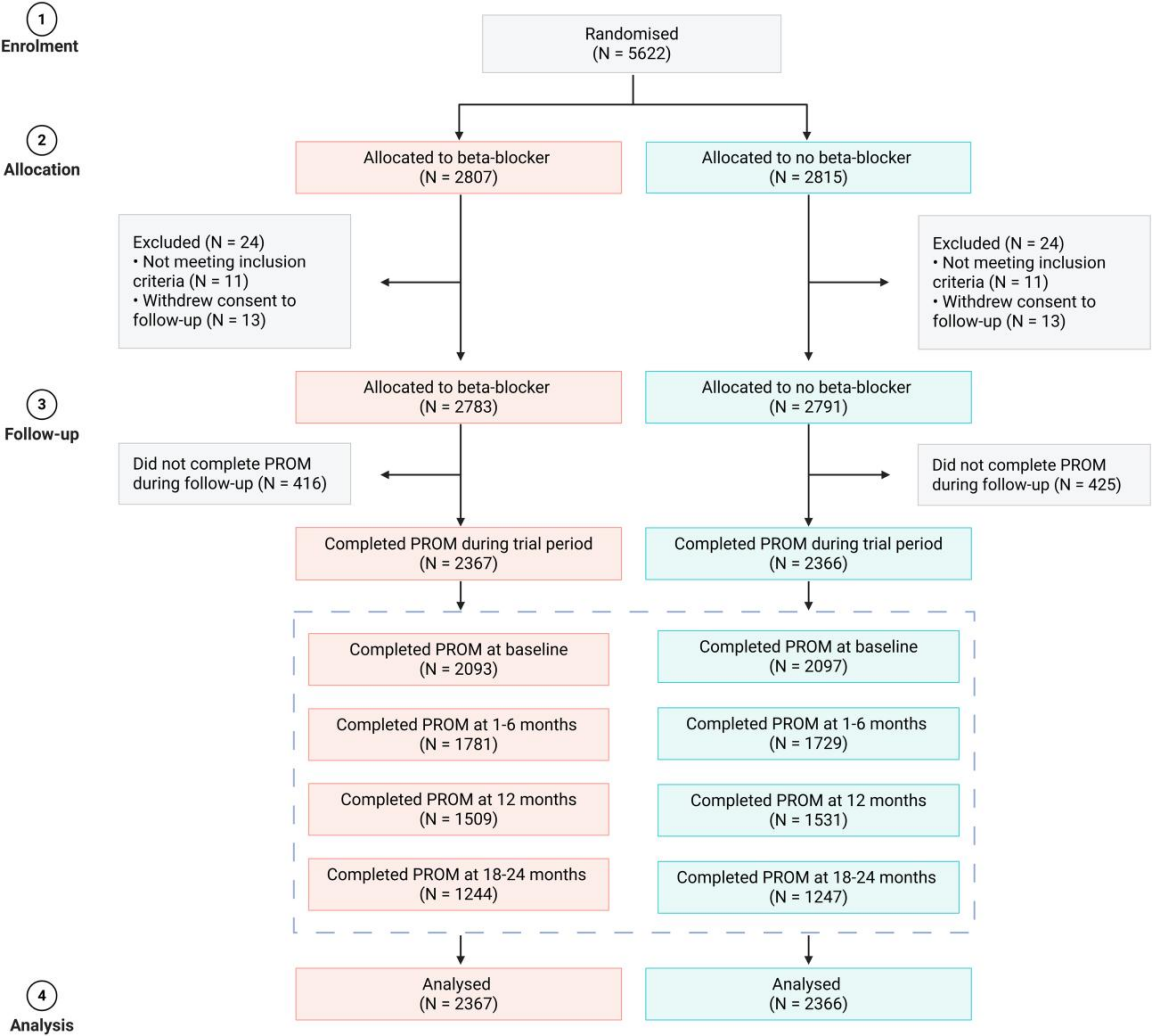
Flow chart of enrolment, randomization, and follow-up. PROM, patient-reported outcome measure

A flow chart depicting the recruitment process and patient attrition is presented in *Figure 1*. Out of 5622 randomized patients, 841 (15.1%) did not complete any PROMs during the trial period and were excluded from the analyses. Age, sex, and other clinical characteristics at baseline were comparable between PROM responders and non-responders (see Supplementary data online, *Table S1*).

Out of 4733 participants who completed PROMs at least once during the study period, 4190 (88.5%) responded to HADS at baseline and 3040 (64.2%) responded at the 12-month follow-up (*Figure 1*). In total, 1831 (89.4%) and 1236 (60.3%) participants from the BETAMI cohort completed PHQ-2 at baseline and at 12-month follow-up, respectively.

The baseline characteristics were similar between the two groups (*Table 1*). Metoprolol was the most common beta-blocker, prescribed to 95.6% of patients randomized to beta-blockers, with a median dose of 50 mg.

**Table 1:** Baseline characteristics of the study population

	All (*N* = 4733)	Beta-blocker (*N* = 2367)	No beta-blocker (*N* = 2366)
Age, years, median (IQR)	62 (55–70)	63 (55–70)	62 (55–70)
Female sex, *n* (%)	980 (20.7)	496 (21.0)	484 (20.5)
University level education, *n* (%)	1472 (33.1)	739 (33.2)	733 (33.1)
Country, *n* (%)			
Norway	2049 (43.3)	1025 (43.3)	1024 (43.3)
Denmark	2684 (56.7)	1342 (56.7)	1342 (56.7)
Cardiovascular risk factors			
Current smoker, *n* (%)	1252 (27.1)	637 (27.6)	615 (26.7)
Body mass index, kg/m^2^, median (IQR)	27.6 (25.1–30.7)	27.5 (25.1–30.5)	27.7 (25.0–30.8)
Hypertension, *n* (%)	1950 (41.2)	960 (40.6)	990 (41.8)
Diabetes, *n* (%)	562 (11.9)	261 (11.0)	301 (12.7)
Low-density lipoprotein cholesterol, mmol/L, median (IQR)	3.3 (2.5–4.1)	3.4 (2.6–4.13)	3.3 (2.5–4.1)
Previous cardiovascular disease, *n* (%)			
Coronary artery disease	480 (9.7)	231 (9.3)	249 (10.0)
Peripheral artery disease	139 (2.9)	66 (2.8)	73 (3.1)
Stroke	117 (2.4)	58 (2.4)	59 (2.5)
Atrial fibrillation	95 (2.0)	42 (1.8)	53 (2.2)
Previous medication, n (%)			
Antidepressant medication ≤5 years prior^a^	329 (12.3)	172 (12.8)	157 (11.7)
Anxiolytic medication ≤ 5 years prior^a^	93 (3.5)	45 (3.4)	48 (3.6)
Prior beta-blocker therapy	405 (8.6)	200 (8.4)	205 (8.7)
Index MI, *n* (%)			
STEMI	2303 (48.7)	1154 (48.8)	1149 (48.6)
LVEF ≥50%	3645 (77.1)	1823 (77.2)	1822 (77.0)
In-hospital treatment, *n* (%)			
PCI	4333 (91.7)	2177 (92.1)	2156 (91.4)
CABG	101 (2.1)	46 (2.0)	55 (2.3)
No revascularization	325 (6.9)	162 (6.8)	163 (6.9)
Symptoms of depression and anxiety^b^			
HADS-D, median (IQR)	2 (0–4)	2 (0–4)	2 (0–4)
HADS-D score ≥ 8, *n* (%)	419 (10.0)	203 (9.7)	216 (10.3)
HADS-A, median (IQR)	3 (1–6)	3 (1–6)	3 (1–6)
HADS-A score ≥ 8, *n* (%)	740 (17.7)	368 (17.6)	372 (17.7)
PHQ-2, median (IQR)	1 (0–2)	1 (0–2)	0 (0–2)
PHQ-2 score ≥ 3, *n* (%)	146 (9.0)	74 (9.1)	72 (8.8)
Medication at discharge, *n* (%)			
Aspirin	4425 (93.5)	2202 (93.0)	2223 (94.0)
P2Y_12_ receptor blocker	4525 (95.6)	2244 (94.8)	2281 (96.4)
Anticoagulants	182 (3.8)	85 (3.6)	97 (4.1)
ACE inhibitor or ARB	1899 (40.1)	959 (40.5)	940 (39.7)
Statin	4552 (96.2)	2267 (95.8)	2285 (96.6)
Ezetimibe	618 (13.1)	302 (12.8)	316 (13.4)
Metoprolol^c^	-	2262 (95.6)	-

IQR, interquartile range; MI, myocardial infarction; STEMI, ST-elevation myocardial infarction; LVEF, left ventricular ejection fraction; ACE, angiotensin-converting enzyme; ARB, angiotensin receptor blocker; PCI, percutaneous coronary intervention; CABG, coronary artery bypass graft; HADS-D, Hospital Anxiety and Depression Scale depression subscale; HADS-A, Hospital Anxiety and Depression Scale anxiety subscale; PHQ-2, Patient Health Questionnaire-2.

^a^Among DANBLOCK participants.

^b^Numbers among 4190 participants who responded to HADS at baseline and 1630 participants in BETAMI responding to PHQ-2 at baseline.

^c^Median metoprolol dose 50 mg/day.

The median HADS-D score at baseline was 2 (IQR: 0–4) in both groups. Of these, 10.0% had baseline HADS-D scores ≥8 indicating clinically significant symptoms of depression. The median HADS-A score was 3 (IQR: 1–6) in the two groups, with 17.7% being clinically significant symptoms of anxiety.

### Intention-to-treat analysis

**Figure 2:**
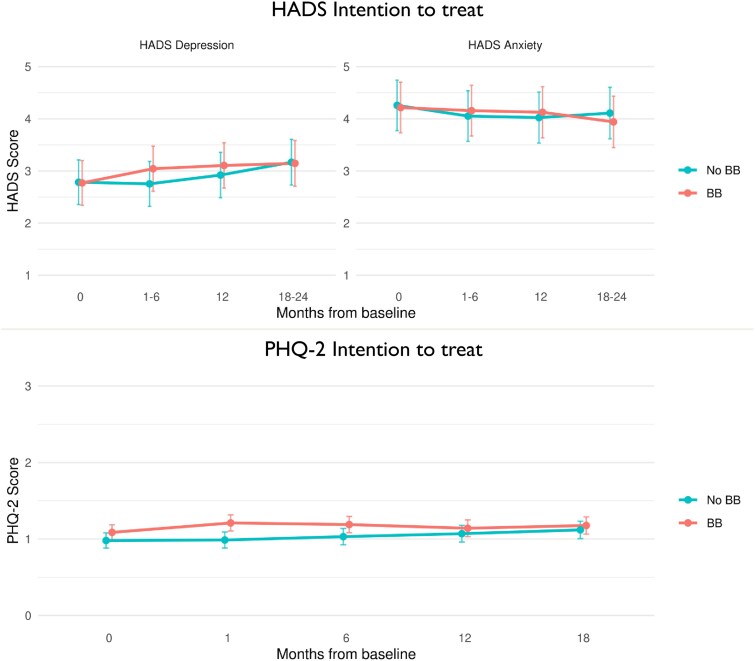
Effect of beta-blocker therapy on symptoms of depression and anxiety. BB, beta-blocker; HADS, Hospital Anxiety and Depression Scale; PHQ-2, Patient Health Questionnaire-2

At 12-month follow-up, patients randomized to the beta-blocker group exhibited a negligible higher increase from baseline to 12 months in HADS-D score compared with the no beta-blocker group (between-group difference in change: 0.20, 95% CI 0.00–0.39) (*Table 2* and *Figure 2*). At 1–6-month follow-up, the difference was 0.30 (95% CI 0.12–0.49), while at 18–24-month follow-up it was −0.01 (95% CI −0.22–0.20). Changes in continuous score of PHQ-2 showed no significant difference in symptoms of depression between the beta-blocker and no beta-blocker groups (*Figure 2* and *Table 2*).

**Table 2:** Estimated difference in change in symptoms of depression and anxiety between the beta-blocker and no beta-blocker group

	*β* (95% CI)			
	1–6 months		12 months	18–24 months
HADS-D	0.30 (0.12, 0.49)		0.20 (−0.00, 0.39)	−0.01 (−0.22, 0.20)
HADS-A	0.15 (−0.06, 0.35)		0.14 (−0.06, 0.35)	−0.13 (−0.35, 0.10)

	1 month	6 months	12 months	18 months
PHQ-2	0.12 (−0.01, 0.25)	0.05 (−0.08, 0.18)	−0.04 (−0.17, 0.10)	−0.05 (−0.19, 0.09)

	OR (95% CI)			
	1–6 months		12 months	18–24 months
HADS-D score ≥ 8	1.47 (0.86, 2.52)		1.40 (0.84, 2.36)	0.78 (0.46, 1.32)
HADS-A score ≥ 8	1.34 (0.84, 2.13)		1.15 (0.73, 1.81)	0.83 (0.50, 1.37)

	1 month	6 months	12 months	18 months
PHQ-2 score ≥ 3	2.18 (0.99, 4.82)	2.09 (0.86, 5.08)	0.68 (0.27, 1.73)	0.80 (0.32, 1.99)

All patients in the study population included in analyses of HADS-A and HADS-D and all patients in the BETAMI cohort included in analyses of PHQ-2.

CI, confidence interval; OR, odds ratio, HADS-D, Hospital Anxiety and Depression Scale depression subscale; HADS-A, Hospital Anxiety and Depression Scale anxiety subscale; PHQ-2, Patient Health Questionnaire-2.

For anxiety, no significant difference in symptoms was observed between the two groups. The between-group differences in changes in HADS-A from baseline were 0.15 (95% CI −0.06–0.35) at 1–6 months, 0.14 (95% CI −0.06–0.35) at 12-month follow-up, and −0.13 (95% CI −0.35–0.10) at 18–24 months (*Table 2* and *Figure 2*).

There was no significant effect of beta-blocker use on clinically significant symptoms of depression or anxiety (*Table 2*). At 12 months, the estimated ORs for depression (beta-blockers vs no beta-blockers) were 1.40 (95% CI 0.84–2.36), evaluated by HADS-D, and 0.68 (95% CI 0.27–1.73), evaluated by PHQ-2. The estimated OR for anxiety at 12 months was 1.15 (95% CI 0.73–1.81). Additional details on scores and number of patients with clinically significant symptoms of depression and anxiety are provided in the Supplementary data online, *Tables S2* and *S3*. The estimated effect of beta-blocker therapy on each individual item of HADS and PHQ-2 revealed no significant discrepancies (see Supplementary data online, *Figure S2*).

### Adherence to assigned group

Self-reported adherence to the assigned group was 88.8% in the beta-blocker group and 89.1% in the no beta-blocker group at 1–6-months, dropping to 80.0% and 87.8% at 12 months and 74.0% and 90.4% at 18 months, respectively (see Supplementary data online, *Table S4*). In a sensitivity analysis, in which patients were assigned to either the beta-blocker group or no beta-blocker group based on self-reported adherence, the results mimicked the main analyses (see Supplementary data online, *Figure S1* and *Table S5*).

### Subgroup analyses

**Figure 3:**
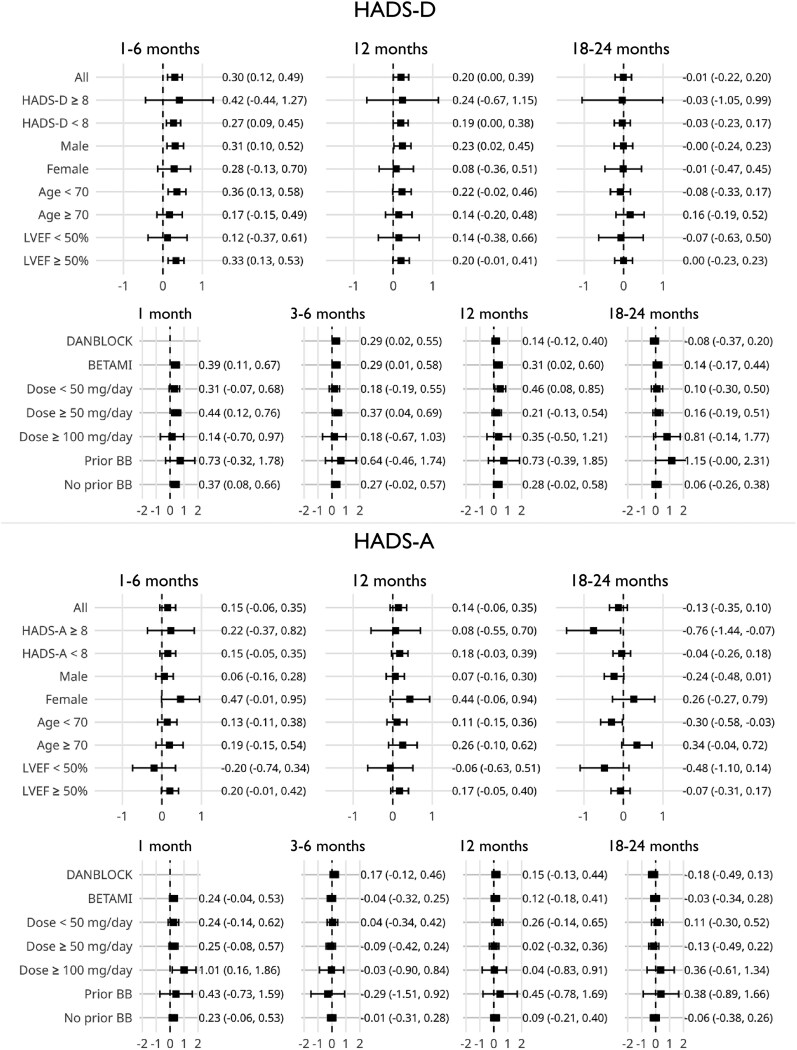
Subgroup analyses of symptoms of depression and anxiety. The figure shows the between-group difference in change in symptoms from baseline to each follow-up in subgroups of the study population. Positive values are an increase in the beta-blocker (BB) group compared with the no BB group. At baseline, 3450 patients were under 70 years of age, while 1283 were 70 years of age or older. Analyses on metoprolol dose and prior BB only in the BETAMI cohort. Number of patients in the BB arm with dose <50 mg/day is 430, ≥50 mg/day is 595, and ≥100 mg/days 52. In BETAMI, 155 patients had prior BB and 1894 no prior BB. CI, Confidence interval; HADS-A, Hospital Anxiety and Depression Scale anxiety subscale; HADS-D, Hospital Anxiety and Depression Scale depression subscale; LVEF, left ventricular ejection fraction; PHQ-2, Patient Health Questionnaire-2

The results were generally consistent across prespecified subgroups, including age, sex, baseline symptoms of anxiety and depression, country, LVEF, beta-blocker dosage, and prior beta-blocker therapy (*Figure 3*). *Post hoc* analyses revealed no clinically relevant differences in scores of anxiety or depression scores among patients treated with metoprolol at a dose of 100 mg/day or higher compared with patients treated with lower doses.

## Discussion

In this prespecified sub-study of patients hospitalized with an MI without heart failure, we did not find any effect of long-term beta-blocker therapy on symptoms of depression or anxiety during the 2-year follow-up period (*Structured Graphical Abstract*). The results appear to be independent of adherence to allocated treatment and consistent across all predefined subgroups. These findings from an adequately powered study provide reassurance that beta-blocker therapy in a moderate dose can be safely used in the post-MI population without increasing the risk of depression or anxiety.

Observational studies have suggested an association between beta-blockers and symptoms of depression.^23–25^ Some of these studies are registry-based and draw conclusions from the simultaneous use of antidepressant medications and beta-blockers,^23,25^ while others have applied screening with questionnaires.^24^ However, observational studies are subject to unmeasured confounding factors such as confounding by indication. Among the early randomized controlled trials shedding light on this question, only one has uncovered an association.^26^ Post-MI patients treated with the non-selective beta-blocker sotalol administered at a dosage 320 mg/day had an almost fivefold increase in the risk of depression compared with placebo (2.4% vs 0.5%).^26^ However, this dosage corresponds to a metoprolol equivalent of 400 mg/day, twice the maximum dose used today. Other trials have failed to reproduce this finding, and meta-analyses of placebo-controlled trials have found no association between beta-blocker therapy and symptoms of depression.^15,27,28^

In later years, the importance of proper methods for surveillance and reporting of side effects has been acknowledged.^29^ Earlier randomized trials examining beta-blocker side effects mainly relied on passive surveillance methods without using standardized and validated instruments. This approach may introduce underreporting bias,^13,15,27^ thus raising the question of the validity and reliability of previous results. The critics also point to the exclusion of patients with a history of depression, which may prevent trials from capturing the patients who are most prone to depression as a side effect.^30^ In the BETAMI-DANBLOCK trial, a history of depression or anxiety was not among the exclusion criteria, and 12.3% of the DANBLOCK population had a history of antidepressant use. Furthermore, 84.9% of the total trial population responded to commonly used questionnaires. Altogether, this minimizes the selection of participants and thus contributes to generalizability to the post-MI population.

The negligible larger change in HADS depression scores of 0.20 (95% CI 0.00–0.39) observed in the beta-blocker arm in our study within the first 12 months is far below the established minimal clinically important difference of 1.7 points.^19^ The changes in HADS depression score are smaller (0.2 vs 0.5 points) than observed in a recent sub-study from the REDUCE-AMI trial.^31^ However, the sample in that study was small and selected (*N* = 806, 16% of the total population) and biased by higher HADS depression score in the beta-blocker arm at baseline. In line with our results, they found no effect of beta-blocker therapy on scores of anxiety.^31,32^ In the ABYSS trial, a small subgroup of participants (*N* = 440, 12% of the total population) completed screening questions regarding their feelings of depression and worry at the 6- and 12-month follow-ups. The results indicated comparable levels of depression and worry among patients who interrupted beta-blocker therapy and those who continued treatment.^10^ Our study expands on these recent studies, providing results for a significantly larger sample of patients ensuring adequate power, well-balanced baseline characteristics, and enabling analyses also on sub-groups of patients. Contrary to the REDUCE-AMI trial, we have also included the subgroup of patients with mildly reduced LVEF. Because recent evidence suggests a favourable effect of beta-blockers in this subgroup, elucidating the incidence of their side effects is especially important for tailoring treatment guidelines.^33^

Depression is associated with non-adherence to cardiovascular drug therapy.^34^ Therefore, one could expect that patients who developed symptoms of depression as a side effect quit their treatment with a subsequent normalisation of symptoms. However, our sensitivity analyses incorporating adherence support the main results. This indicates that potential effects of beta-blocker therapy have not been diluted by non-adherence. The PHQ-2 and HADS questionnaires are both used to assess symptoms of depression, but they differ in their structure and content, and do not fully identify the same cases.^35^ Consequently, it is a strength that the results were similar across both assessment methods. Finally, our results appear consistent in all important high-risk subgroups including the elderly, women, and those with and without pre-existing symptoms of depression and anxiety at baseline.

### Limitations

Some limitations must be acknowledged. First, as an open-label trial, the patient-reported outcomes might be prone to bias due to participants’ expectations to the treatment received. Though it is acknowledged that patients’ expectations can influence reported side effects, specific expectations among the participants are expected to be small as the patient completed several different questionnaires simultaneously. Second, with the exception of the baseline and 12-month follow-up, the BETAMI and DANBLOCK trials employed different time points for collecting patient-reported data. Nevertheless, this potential limitation was addressed through the use of mixed models for data analysis. Third, even though other tools to evaluate symptoms of anxiety and depression exists, HADS and PHQ-2 are commonly used and reliable tools, capturing some of the central symptoms of depression and anxiety such as depressed mood, anhedonia, worry, and fearfulness.^17,21,36^ To explore if any specific items were affected by beta-blocker therapy, we also conducted an individual item analysis without detecting any significant discrepancies. Fourth, self-reported adherence measures have previously been shown to be unreliable, often overestimating adherence to cardiovascular medications.^37^ Unfortunately, data on long-term adherence from the national drug registries were not available for the entire population. On the other hand, prescription refill data are also affected by measurements errors due to the inability to capture whether collected prescriptions have actually been consumed. Fifth, in BETAMI-DANBLOCK, almost all patients were prescribed metoprolol in a modest median dose (50 mg), and one should be cautious in generalizing the results to other beta-blocker classes or doses. However, *post hoc* analyses indicate no clinically relevant differences in HADS-D and HADS-A scores among the subgroup of patients treated with metoprolol doses of 100 mg/day or more compared with those treated with lower doses. Thus, our data did not suggest any dose-dependent response to metoprolol. Furthermore, the treatment dosage was at the discretion of the treating cardiologists and reflects contemporary clinical practice. Sixth, there is potential for participation bias to affect the results of our study, particularly if drop-out rates are associated with randomization or symptoms of depression or anxiety. The most significant impediment would be if some patients experiencing depressive or anxious symptoms chose to stop completing questionnaires. However, the high completion rate on the questionnaires at baseline and during follow-up suggests that this is unlikely to significantly influence our findings. Finally, future studies are needed to evaluate the effect of beta-blocker therapy on other potentially relevant neuropsychological symptoms such as fatigue and insomnia.

## Conclusions

In this prespecified sub-study of a randomized open-label trial, beta-blocker therapy at moderate doses had no clinically meaningful impact on symptoms of depression and anxiety in post-MI patients with mildly reduced or preserved LVEF.

## Supplementary Material

ehag200_Supplementary_Data

## Declarations

### Data Availability

According to Danish and Norwegian legislation, the Data Protection Authorities, and the Committees of Ethics, we are not allowed to share original study data publicly. Data from official registries have been used in this publication. The interpretation and reporting of these data are the sole responsibility of the authors, and no endorsement by the registers is intended nor should be inferred.

### Pre-registered Clinical Trial Number

The pre-registered clinical trial numbers are NCT03778554 and NCT03646357.

## Appendix

### Self-reported screening questions concerning adherence

Adherence to randomization in BETAMI was identified based on the response to the self-reported screening question ‘Are you using medications of the beta-blocker type (e.g. Selo-zok, Emconcor, Metoprolol, Carvedilol, Atenolol, Tenormin, Bisoprolol, Uniloc)’. Patients responding ‘yes’ were classified as adherent if randomized to beta-blockers, and patients responding ‘no’ were classified as adherent if randomized to no beta-blocker.

Adherence to randomization in DANBLOCK was identified from two different screening questions dependent on randomization group. Patients randomized to beta-blockers responding ‘no’ to the question ‘Have you stopped permanently taking beta-blockers (i.e. have you NOT taken beta-blockers in the last 3 weeks)?’ were classified as adherent. Patients randomized to no beta-blockers responding ‘no’ to the question ‘Has your physician or cardiologist prescribed beta-blockers for you?’ were classified as adherent.

## References

[1] Feng HP, Chien WC, Cheng WT, Chung CH, Cheng SM, Tzeng WC. Risk of anxiety and depressive disorders in patients with myocardial infarction: a nationwide population-based cohort study. Medicine (Baltimore) 2016;95:e4464. 10.1097/MD.0000000000004464 27559951 PMC5400317

[2] Leissner P, Held C, Humphries S, Rondung E, Olsson EMG. Association of anxiety and recurrent cardiovascular events: investigating different aspects of anxiety. Eur J Cardiovasc Nurs 2024;23:720–7. 10.1093/eurjcn/zvae036 38518740

[3] Garrels E, Kainth T, Silva B, Yadav G, Gill G, Salehi M, et al Pathophysiological mechanisms of post-myocardial infarction depression: a narrative review. Front Psychiatry 2023;14:1225794. 10.3389/fpsyt.2023.1225794 37599890 PMC10436342

[4] Byrne RA, Rossello X, Coughlan JJ, Barbato E, Berry C, Chieffo A, et al 2023 ESC guidelines for the management of acute coronary syndromes: developed by the task force on the management of acute coronary syndromes of the European Society of Cardiology (ESC). Eur Heart J 2023;44:3720–826. 10.1093/eurheartj/ehad191 37622654

[5] Rao SV, O'Donoghue ML, Ruel M, Rab T, Tamis-Holland JE, Alexander JH, et al 2025 ACC/AHA/ACEP/NAEMSP/SCAI guideline for the management of patients with acute coronary syndromes: a report of the American College of Cardiology/American Heart Association joint committee on clinical practice guidelines. Circulation 2025;151:e771–862. 10.1161/CIR.0000000000001328 40014670

[6] Cataldo Miranda P, Gasevic D, Trin C, Stub D, Zoungas S, Kaye DM, et al Beta-blocker therapy after myocardial infarction. JACC Adv 2025;4:101582. 10.1016/j.jacadv.2024.101582 39889325 PMC11834082

[7] Yndigegn T, Lindahl B, Mars K, Alfredsson J, Benatar J, Brandin L, et al Beta-blockers after myocardial infarction and preserved ejection fraction. N Engl J Med 2024;390:1372–81. 10.1056/NEJMoa2401479 38587241

[8] Ibanez B, Latini R, Rossello X, Dominguez-Rodriguez A, Fernández-Vazquez F, Pelizzoni V, et al Beta-blockers after myocardial infarction without reduced ejection fraction. N Engl J Med 2025;393:1889–900. 10.1056/NEJMoa2504735 40888702

[9] Munkhaugen J, Kristensen Anna Meta D, Halvorsen S, Holmager T, Olsen Michael H, Bakken A, et al Beta-blockers after myocardial infarction in patients without heart failure. N Engl J Med 2025;393:1901–11. 10.1056/NEJMoa2505985 40888716

[10] Silvain J, Cayla G, Ferrari E, Range G, Puymirat E, Delarche N, et al Beta-blocker interruption or continuation after myocardial infarction. N Engl J Med 2024;391:1277–86. 10.1056/NEJMoa2404204 39213187

[11] Frishman WH, Furberg CD, Friedewald WT. Beta-adrenergic blockade for survivors of acute myocardial infarction. N Engl J Med 1984;310:830–7. 10.1056/NEJM198403293101306 6142420

[12] Ahmed AI, van Mierlo P, Jansen P. Sleep disorders, nightmares, depression and anxiety in an elderly patient treated with low-dose metoprolol. Gen Hosp Psychiatry 2010;32:646.e5–7. 10.1016/j.genhosppsych.2010.04.008 21112460

[13] Waal HJ . Propranolol-induced depression. Br Med J 1967;2:50. 10.1136/bmj.2.5543.50 PMC1841098 6021004

[14] Yilmaz MB, Erdem A, Yalta K, Turgut OO, Yilmaz A, Tandogan I. Impact of beta-blockers on sleep in patients with mild hypertension: a randomized trial between nebivolol and metoprolol. Adv Ther 2008;25:871–83. 10.1007/s12325-008-0087-x 18758699

[15] Ko DT, Hebert PR, Coffey CS, Sedrakyan A, Curtis JP, Krumholz HM. Beta-blocker therapy and symptoms of depression, fatigue, and sexual dysfunction. JAMA 2002;288:351–7. 10.1001/jama.288.3.351 12117400

[16] Kristensen AMD, Munkhaugen J, Halvorsen S, Olsen MH, Bakken A, Sehested TSG, et al The Danish-Norwegian randomized trial on beta-blocker therapy after myocardial infarction: design, rationale, and baseline characteristics. Eur Heart J Cardiovasc Pharmacother 2024;10:175–83. 10.1093/ehjcvp/pvad093 38017624

[17] Bjelland I, Dahl AA, Haug TT, Neckelmann D. The validity of the hospital anxiety and depression scale: an updated literature review. J Psychosom Res 2002;52:69–77. 10.1016/S0022-3999(01)00296-3 11832252

[18] Zigmond AS, Snaith RP. The hospital anxiety and depression scale. Acta Psychiatr Scand 1983;67:361–70. 10.1111/j.1600-0447.1983.tb09716.x 6880820

[19] Lemay KR, Tulloch HE, Pipe AL, Reed JL. Establishing the minimal clinically important difference for the hospital anxiety and depression scale in patients with cardiovascular disease. J Cardiopulm Rehabil Prev 2019;39:E6–e11. 10.1097/HCR.0000000000000379 30489438

[20] Kroenke K, Spitzer RL, Williams JB. The patient health questionnaire-2: validity of a two-item depression screener. Med Care 2003;41:1284–92. 10.1097/01.MLR.0000093487.78664.3C 14583691

[21] Löwe B, Kroenke K, Gräfe K. Detecting and monitoring depression with a two-item questionnaire (PHQ-2). J Psychosom Res 2005;58:163–71. 10.1016/j.jpsychores.2004.09.006 15820844

[22] Mitchell AJ, Yadegarfar M, Gill J, Stubbs B. Case finding and screening clinical utility of the patient health questionnaire (PHQ-9 and PHQ-2) for depression in primary care: a diagnostic meta-analysis of 40 studies. BJPsych Open 2016;2:127–38. 10.1192/bjpo.bp.115.001685 27703765 PMC4995584

[23] Avorn J, Everitt DE, Weiss S. Increased antidepressant use in patients prescribed beta-blockers. JAMA 1986;255:357–60. 10.1001/jama.1986.03370030077031 2867235

[24] Ringoir L, Pedersen SS, Widdershoven JW, Pouwer F, Keyzer JM, Romeijnders AC, et al Beta-blockers and depression in elderly hypertension patients in primary care. Fam Med 2014;46:447–53. 24911300

[25] Thiessen BQ, Wallace SM, Blackburn JL, Wilson TW, Bergman U. Increased prescribing of antidepressants subsequent to beta-blocker therapy. Arch Intern Med 1990;150:2286–90. 10.1001/archinte.1990.00390220044009 1978648

[26] Julian DG, Jackson FS, Prescott RJ, Szekely P. Controlled trial of sotalol for one year after myocardial infarction. Lancet 1982;319:1142–7. 10.1016/S0140-6736(82)92225-5 6122937

[27] Riemer TG, Villagomez Fuentes LE, Algharably EAE, Schäfer MS, Mangelsen E, Fürtig MA, et al Do β-blockers cause depression?: systematic review and meta-analysis of psychiatric adverse events during β-blocker therapy. Hypertension 2021;77:1539–48. 10.1161/HYPERTENSIONAHA.120.16590 33719510

[28] Zhang L, Bao Y, Tao S, Zhao Y, Liu M. The association between cardiovascular drugs and depression/anxiety in patients with cardiovascular disease: a meta-analysis. Pharmacol Res 2022;175:106024. 10.1016/j.phrs.2021.106024 34890773

[29] Bent S, Padula A, Avins AL. Brief communication: better ways to question patients about adverse medical events: a randomized, controlled trial. Ann Intern Med 2006;144:257–61. 10.7326/0003-4819-144-4-200602210-00007 16490911

[30] Steffensmeier JJ, Ernst ME, Kelly M, Hartz AJ. Do randomized controlled trials always trump case reports? A second look at propranolol and depression. Pharmacotherapy 2006;26:162–7. 10.1592/phco.26.2.162 16466322

[31] Leissner P, Mars K, Humphries S, Karlström P, Yndigegn T, Jernberg T, et al Short- and long-term effects of beta-blockers on symptoms of anxiety and depression in patients with myocardial infarction and preserved left ventricular function: a pre-specified quality of life sub-study from the REDUCE-AMI trial. Eur Heart J Acute Cardiovasc Care 2024;13:789–97. 10.1093/ehjacc/zuae112 39422765 PMC11638855

[32] Leissner P, Mars K, Humphries S, Jernberg T, Held C, Hofmann R, et al A randomized controlled trial of beta-blockers effects on cardiac anxiety. Gen Hosp Psychiatry 2025;94:26–32. 10.1016/j.genhosppsych.2025.02.010 39983429

[33] Rossello X, Prescott EIB, Kristensen AMD, Latini R, Fuster V, Fagerland MW, et al β blockers after myocardial infarction with mildly reduced ejection fraction: an individual patient data meta-analysis of randomised controlled trials. Lancet 2025;406:1128–37. 10.1016/S0140-6736(25)01592-2 40897190

[34] Jones D, Yue J, Nguyen T, Ahmad F, Chow C. The impact of depression on medication nonadherence in coronary artery disease. Heart Lung Circ 2024;33:S540. 10.1016/j.hlc.2024.06.905

[35] Hansson M, Chotai J, Nordstöm A, Bodlund O. Comparison of two self-rating scales to detect depression: HADS and PHQ-9. Br J Gen Pract 2009;59:e283–8. 10.3399/bjgp09X454070 19761655 PMC2734374

[36] Christensen AV, Dixon JK, Juel K, Ekholm O, Rasmussen TB, Borregaard B, et al Psychometric properties of the Danish hospital anxiety and depression scale in patients with cardiac disease: results from the DenHeart survey. Health Qual Life Outcomes 2020;18:9. 10.1186/s12955-019-1264-0 31910859 PMC6947856

[37] Kristiansen O, Sverre E, Peersen K, Fagerland MW, Gjertsen E, Gullestad L, et al The relationship between directly measured statin adherence, self-reported adherence measures and cholesterol levels in patients with coronary heart disease. Atherosclerosis 2021;336:23–9. 10.1016/j.atherosclerosis.2021.09.020 34610521

