# Supplementary material for: Beta-blockers, depression, and anxiety after myocardial infarction: the BETAMI-DANBLOCK trial
Source: Eur Heart J. 2026 Mar 25;47(25):3270–80. doi: 10.1093/eurheartj/ehag200 (PMC13318420; doi:10.1093/eurheartj/ehag200)
Supplement: ehag200_Supplementary_Data [file ehag200_supplementary_data.docx]

**Supplementary Table 1.** Baseline characteristics of patients responding and not responding to the PROMS questionnaire

|  | **Responders (N = 4733)** | **Non responders (N = 841)** |
| --- | --- | --- |
| Age, median (IQR), y | 62 (55-70) | 63 (55-72) |
| Sex, women, no (%) | 980 (20.7) | 182 (21.6) |
| University level education, no (%) | 1472 (33.1) | 31 (26.3) |
| Country, no (%) |  |  |
| **-** Norway | 2049 (43.3) | 818 (97.3) |
| **-** Denmark | 2684 (56.7) | 23 (2.7) |
| **Cardiovascular risk factors** |  |  |
| Current smoker, no (%) | 1252 (27.1) | 33 (24.6) |
| Body mass index, median (IQR) | 27.6 (25.1-30.7) | 27.6 (25.0-30.6) |
| Hypertension, no (%) | 1950 (41.2) | 329 (39.2) |
| Diabetes, no (%) | 562 (11.9) | 131 (15.6) |
| Low density lipoprotein cholesterol, median (IQR) | 3.3 (2.5-4.1) | 3.4 (2.6-4.2) |
| **Previous cardiovascular disease, no (%)** |  |  |
| Coronary artery disease | 480 (9.7) | 110 (13.1) |
| Peripheral artery disease | 139 (2.9) | 27 (3.2) |
| Stroke | 117 (2.4) | 39 (4.6) |
| Atrial fibrillation | 95 (2.0) | 14 (1.7) |
| **Previous medication, no (%)** |  |  |
| Prior beta-blocker therapy | 405 (8.6) | 60 (7.1) |
| **Index MI, no (%)** |  |  |
| STEMI | 2303 (48.7) | 343 (40.8) |
| LVEF ≥50% | 3645 (77.1) | 692 (82.3) |
| **In hospital treatment, no (%)** |  |  |
| PCI | 4333 (91.7) | 829 (98.6) |
| CABG | 101 (2.1) | 0 (0.0) |
| No revascularization | 325 (6.9) | 19 (2.3) |
| **Medication at discharge, no (%)** |  |  |
| Aspirin | 4425 (93.5) | 827 (98.3) |
| P2Y_12_ receptor blocker | 4525 (95.6) | 815 (96.9) |
| Anticoagulants | 182 (3.8) | 33 (3.9) |
| ACE inhibitor or ARB | 1899 (40.1) | 397 (47.2) |
| Statin | 4552 (96.2) | 823 (97.9) |
| Ezetimibe | 618 (13.1) | 69 (8.2) |

PROMS, patient reported outcome measures; IQR, interquartile range; STEMI, ST-segment elevation MI; LVEF, left ventricular ejection fraction; PCI, percutaneous coronary intervention; CABG, coronary artery bypass grafting; ACE, angiotensin-converting enzyme; ARB, angiotensin-receptor blocker

**Supplementary Table 2**. Scores and clinically significant symptoms of depression and anxiety during follow-up in the study population per randomisation group

|  | Beta-blocker | No beta-blocker |
| --- | --- | --- |
| HADS-D score, median (IQR) |  |  |
| - Baseline | 2 (0-4) | 2 (0-4) |
| - 1-6 months | 2 (1-5) | 2 (1-4) |
| - 12 months | 2 (1-5) | 2 (0-4) |
| - 18-24 months | 2 (1-5) | 2 (1-4) |
| HADS-D ≥ 8, no (%) |  |  |
| - Baseline | 203/2093 (9.70) | 216/2097 (10.30) |
| - 1-6 months | 186/1781 (10.44) | 151/1729 (8.73) |
| - 12 months | 164/1509 (10.87) | 156/1531 (10.19) |
| - 18-24 months | 136/1244 (10.93) | 146/1247 (11.71) |
| HADS-A score, median (IQR) |  |  |
| - Baseline | 3 (1-6) | 3 (1-6) |
| - 1-6 months | 3 (1-6) | 3 (1-6) |
| - 12 months | 3 (1-6) | 3 (1-6) |
| - 18-24 months | 3 (1-6) | 3 (1-6) |
| HADS-A ≥ 8, no (%) |  |  |
| - Baseline | 368/2093 (17.58) | 372/2097 (17.74) |
| - 1-6 months | 287/1781 (16.11) | 256/1729 (14.81) |
| - 12 months | 249/1509 (16.50) | 239/1531 (15.61) |
| - 18-24 months | 178/1244 (14.31) | 194/1247 (15.56) |
| PHQ-2 score, median (IQR) |  |  |
| - Baseline | 1 (0-2) | 0 (0-2) |
| - 1 month | 1 (0-2) | 1 (0-2) |
| - 6 months | 1 (0-2) | 1 (0-2) |
| - 12 months | 1 (0-2) | 1 (0-2) |
| - 18 months | 1 (0-2) | 1 (0-2) |
| PHQ-2 ≥ 3, no (%) |  |  |
| - Baseline | 74/814 (9.09) | 72/816 (8.82) |
| - 1 month | 65/671 (9.69) | 47/659 (7.13) |
| - 6 months | 47/627 (7.50) | 34/626 (5.43) |
| - 12 months | 31/565 (5.49) | 34/577 (5.89) |
| - 18 months | 36/508 (7.09) | 37/496 (7.46) |

IQR, interquartile range; HADS-D; Hospital Anxiety and Depression Scale depression subscale; HADS-A, Hospital Anxiety and Depression Scale anxiety subscale

**Supplementary Table 3.** Symptoms of depression and anxiety in BETAMI and DANBLOCK assessed by mean HADS-D and HADS-A scores during follow-up

|  | **BETAMI** | | | |
| --- | --- | --- | --- | --- |
|  | HADS Depression  mean (SD), N | | HADS Anxiety  mean (SD), N | |
|  | Beta-blocker | No beta-blocker | Beta-blocker | No beta-blocker |
| Baseline | 3.13 (3.09)  N=770 | 3.15 (3.04)  N=795 | 4.24 (3.58)  N=770 | 4.22 (3.5)  N=795 |
| 1 month | 2.94 (3.24)  N=650 | 2.67 (2.87)  N=642 | 4.10 (3.68)  N=650 | 3.87 (3.55)  N=642 |
| 6 months | 3.19 (3.37)  N=612 | 3.00 (3.37)  N=603 | 4.04 (3.83)  N=612 | 3.98 (3.60)  N=603 |
| 12 months | 3.27 (3.29)  N=546 | 2.89 (3.33)  N=551 | 4.18 (3.79)  N=546 | 3.85 (3.69)  N=551 |
| 18 months | 3.3 (3.47)  N=487 | 3.28 (3.52)  N=484 | 3.95 (3.94)  N=487 | 4.04 (3.73)  N=484 |
|  | **DANBLOCK** | | | |
|  | HADS Depression  mean (SD), N | | HADS Anxiety  mean (SD), N | |
|  | Beta-blocker | No beta-blocker | Beta-blocker | No beta-blocker |
| Baseline | 2.65 (3.23),  N=1323 | 2.65 (3.20),  N=1302 | 3.99 (3.81),  N=1323 | 4.06 (3.82),  N=1302 |
| 3 months | 2.96 (3.28),  N=965 | 2.64 (3.09),  N=914 | 3.90 (3.60),  N=965 | 3.76 (3.54),  N=914 |
| 12 months | 2.86 (3.14),  N=963 | 2.77 (3.22),  N=980 | 3.70 (3.43),  N=963 | 3.63 (3.47),  N=980 |
| 24 months | 2.79 (3.12),  N=757 | 2.85 (3.39),  N=763 | 3.41 (3.35),  N=757 | 3.71 (3.49),  N=763 |

HADS, Hospital Anxiety and Depression Scale; SD, standard deviation.

**Supplementary Table 4**. Self-reported adherence to allocated treatment during follow-up

|  | **All** | **Beta-blocker** | **No beta-blocker** |
| --- | --- | --- | --- |
| Self-reported adherence at 1-6 months ^a^, no (%) | 2501/2812 (88.9) | 1262/1421 (88.8) | 1239/1391 (89.1) |
| Self-reported adherence at 12 months ^b^, no (%) | 2121/2528 (83.9) | 1009/1262 (80.0) | 1112/1266 (87.8) |
| Self-reported adherence at 18-24 months ^c^, no (%) | 1758/2142 (82.1) | 801/1083 (74.0) | 957/1059 (90.4) |

^a^360 missing in beta-blocker group and 388 missing in no beta-blocker group.

^b^247 missing in beta-blocker group and 265 missing in no beta-blocker group.

^c^161 missing in beta-blocker group and 188 missing in no beta-blocker group.

**Supplementary Table 5.** Estimated difference in change in symptoms of depression and anxiety according to treatment allocation among adherent patients

|  | **β (95% CI)** | | **β (95% CI)** | **β (95% CI)** |
| --- | --- | --- | --- | --- |
|  | 1-6 months | | 12 months | 18-24 months |
| HADS-D | 0.33 (0.12, 0.54) | | 0.33 (0.11, 0.55) | 0.13 (-0.10, 0.37) |
| HADS-A | 0.14 (-0.09, 0.37) | | 0.24 (-0.00, 0.48) | 0.03 (-0.22, 0.29) |
|  | 1 month | 6 months | 12 months | 18 months |
| PHQ-2 | 0.11 (-0.02, 0.25) | 0.06 (-0.08, 0.19) | 0.01 (-0.13, 0,16) | -0.01 (-0.17, 0.14) |
|  | **OR (95% CI)** | | **OR (95% CI)** | **OR (95% CI)** |
|  | 1-6 months | | 12 months | 18-24 months |
| HADS-D score ≥ 8 | 2.00 (1.00, 4.00) | | 1.87 (0.96, 3.62) | 1.00 (0.52, 1.94) |
| HADS-A score ≥ 8 | 1.39 (0.77, 2.51) | | 1.22 (0.70, 2.13) | 1.07 (0.57, 2.00) |
|  | 1 month | 6 months | 12 months | 18 months |
| PHQ-2 score ≥ 3 | 2.17 (0.92, 5.13) | 2.27 (0.87, 5.95) | 0.80 (0.29, 2.19) | 1.22 (0.44, 3.41) |

CI, confidence interval; OR, odds ratio; HADS-D, Hospital Anxiety and Depression Scale depression subscale; HADS-A, Hospital Anxiety and Depression Scale anxiety subscale; PHQ-2, Patient Health Questionnaire 2

Note: All patients in the study population self-reporting adherence to treatment allocation included in analyses of HADS-A and HADS-D, and all patients in the BETAMI-cohort self-reporting adherence to treatment allocation included in analyses of PHQ-2.

**Supplementary Figure 1.** Effect of beta-blocker therapy on symptoms of depression and anxiety among adherent patients

| HADS Adherent patients |
| --- |
| 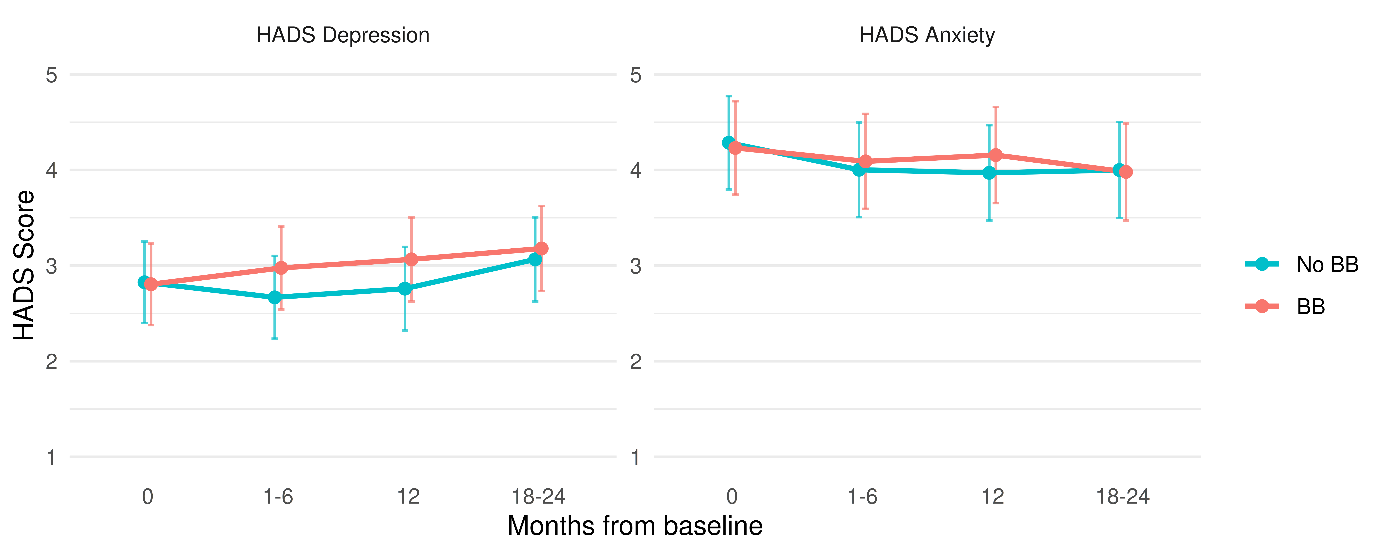 |
| PHQ-2 Adherent patients |
| 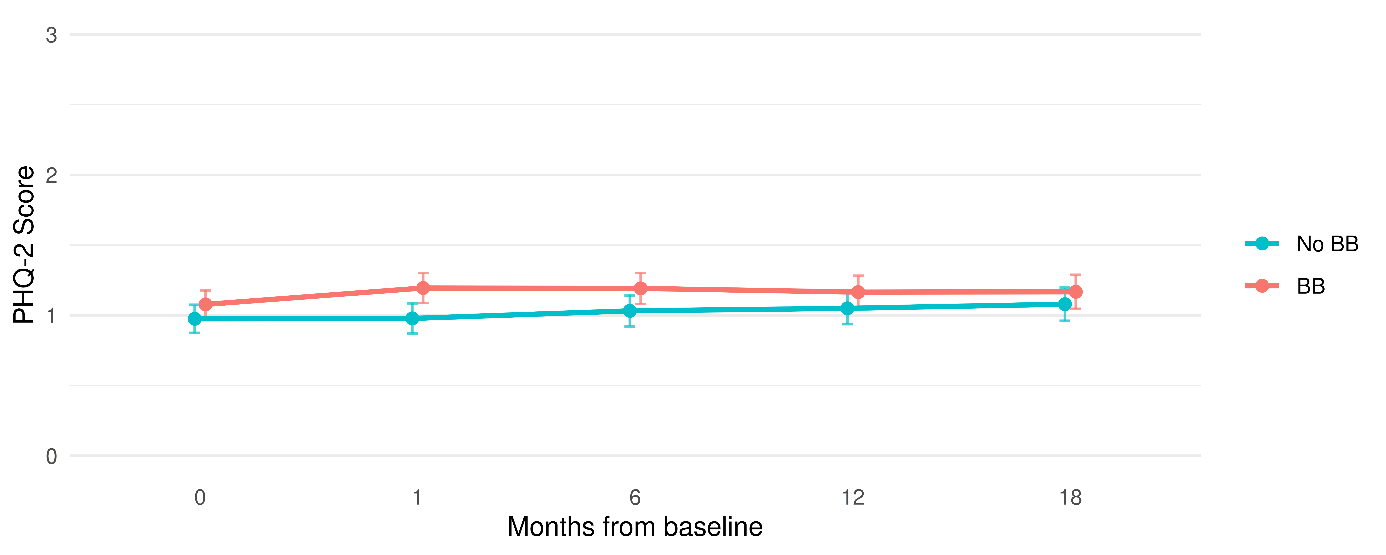 |

HADS, Hospital Anxiety and Depression Scale; PHQ-2, Patient Health Questionnaire 2; BB, beta-blocker

**Supplementary Figure 2.** Estimated difference in each item of HADS and PHQ-2 according to treatment allocation

| 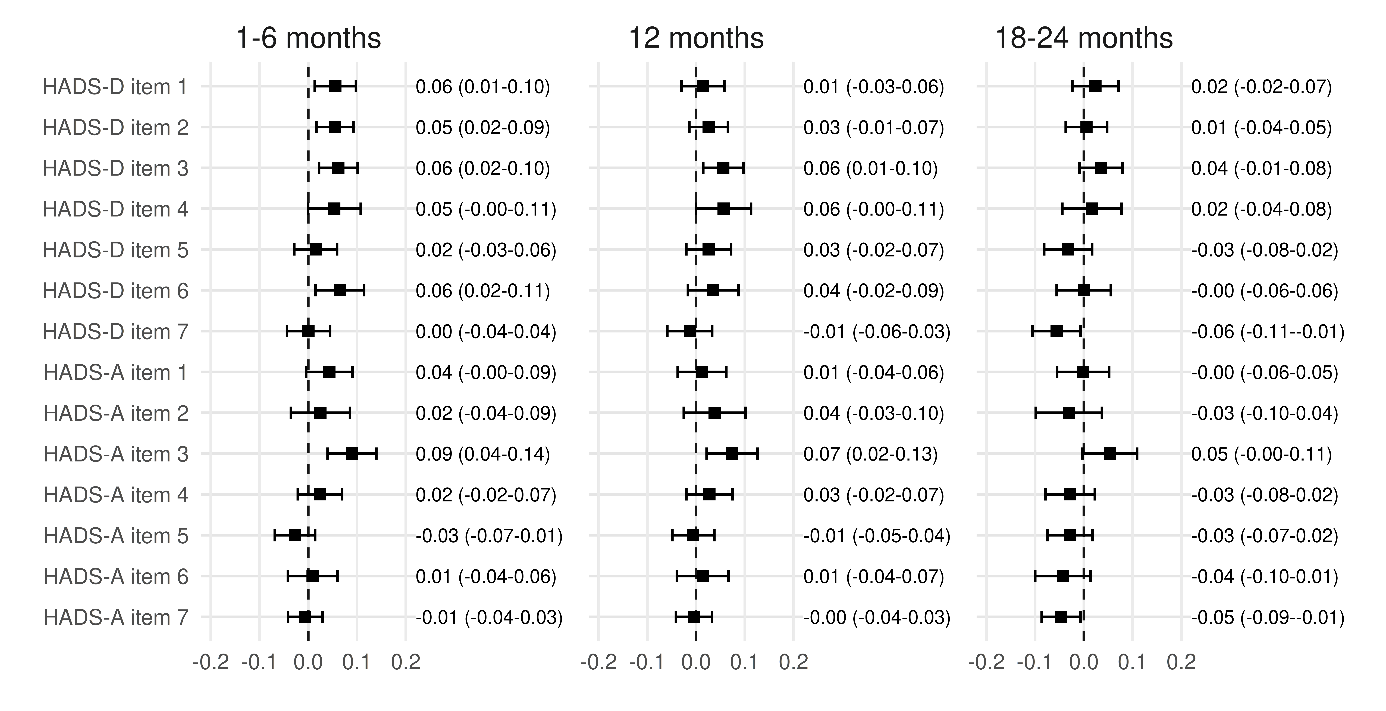 |
| --- |
| 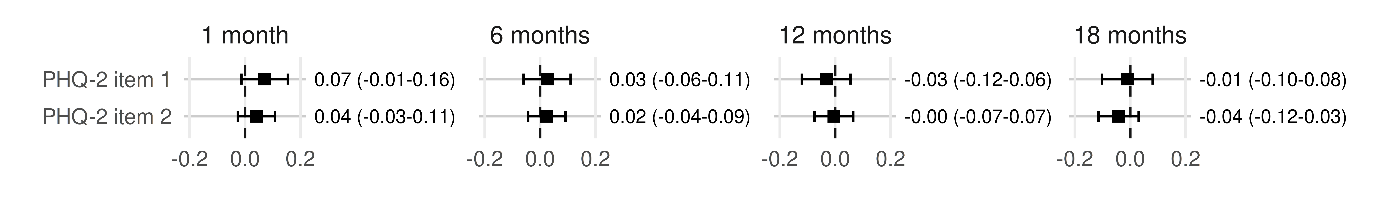 |

HADS-D, Hospital Anxiety and Depression Scale depression subscale; HADS-A, Hospital Anxiety and Depression Scale anxiety subscale; PHQ-2, Patient Health Questionnaire 2

Note: Figure shows the between-group difference in change in symptoms from baseline to each follow-up, on individual items of HADS and PHQ-2. Positive values are an increase in beta-blocker group compared to the no beta-blocker group. All patients in the study population included in analyses of HADS, and all patients in the BETAMI-cohort included in analyses of PHQ-2.
